# Gastrostomy Tube Insertion in Pediatric Patients With Autosomal Recessive Polycystic Kidney Disease (ARPKD): Current Practice

**DOI:** 10.3389/fped.2018.00164

**Published:** 2018-06-04

**Authors:** Kathrin Burgmaier, Joy Brandt, Rukshana Shroff, Peter Witters, Lutz T. Weber, Jörg Dötsch, Franz Schaefer, Djalila Mekahli, Max C. Liebau

**Affiliations:** ^1^Department of Pediatrics, University Hospital of Cologne, Cologne, Germany; ^2^Great Ormond Street Hospital for Children NHS Foundation Trust, London, United Kingdom; ^3^Department of Pediatric Gastroenterology and Hepatology, University Hospitals Leuven, Leuven, Belgium; ^4^Division of Pediatric Nephrology, Center for Pediatric and Adolescent Medicine, University of Heidelberg, Heidelberg, Germany; ^5^Department of Pediatric Nephrology, University Hospitals Leuven, Leuven, Belgium; ^6^PKD Research Group, Department of Development and Regeneration, KU Leuven, University of Leuven, Leuven, Belgium; ^7^Center for Molecular Medicine, University Hospital of Cologne, Cologne, Germany

**Keywords:** ARPKD, congenital hepatic fibrosis, portal hypertension, peritoneal dialysis, *PKHD1*, pediatric polycystic kidney disease

## Abstract

**Introduction:** Autosomal recessive polycystic kidney disease (ARPKD) is a severe hepatorenal disorder of childhood. Early renal disease in ARPKD may require renal replacement therapy and is associated with failure to thrive resulting in a need for nasogastric tube feeding or gastrostomy. In ARPKD patients, the benefit of a gastrostomy in nutrition and growth needs to be weighed against the potential risk of complications of congenital hepatic fibrosis (CHF) and portal hypertension like variceal bleeding. CHF in ARPKD has thus been considered as a relative contraindication for gastrostomy insertion. Yet, data on gastrostomies in pediatric patients with ARPKD is lacking.

**Methods:** We conducted a web-based survey study among pediatric nephrologists, pediatric hepatologists and pediatric gastroenterologists on their opinions on and experiences with gastrostomy insertion in ARPKD patients.

**Results:** 196 participants from 39 countries shared their opinion. 45% of participants support gastrostomy insertion in all ARPKD patients, but portal hypertension is considered to be a contraindication by a subgroup of participants. Patient-specific data was provided for 38 patients indicating complications of gastrostomy that were in principal comparable to non-ARPKD patients. Bleeding episodes were reported in 3/38 patients (7.9%). Two patients developed additional severe complications. Gastrostomy was retrospectively considered as the right decision for the patient in 35/38 (92.1%) of the cases.

**Conclusions:** This report on the results of an online survey gives first insights into the clinical practice of gastrostomy insertion in ARPKD patients. For the majority of participating physicians benefits of gastrostomy insertion retrospectively outweigh complications and risks. More data will be required to lay the foundation for clinical recommendations.

## Introduction

Autosomal recessive polycystic kidney disease (ARPKD) is a rare but severe disorder mainly affecting the liver and the kidneys. The disorder represents one of the leading reasons for pediatric dialysis and kidney-, liver- or combined liver and kidney transplantation. Renal involvement may present very early in life with massively enlarged polycystic kidneys and end stage kidney disease (ESKD), requiring renal replacement therapy (1, 2). Liver involvement due to congenital hepatic fibrosis (CHF) tends to present later in life and is associated with portal hypertension in up to 60% of patients with subsequent development of splenomegaly and collateral circulation (1–3). Esophageal varices have been reported in up to 56% of ARPKD patients with the risk of variceal bleeding (1–3).

Chronic kidney disease (CKD) is associated with growth failure and it has been suggested that ARPKD patients are at a particularly high risk (3, 4). Immaturity and early uremia may affect enteral feeding. Severe ARPKD is also accompanied by massive kidney enlargement and pulmonary hypoplasia. Abdominal distension and ventilation may complicate nutrition resulting in a need for persistent nasogastric feeding.

Concerns have been raised concerning gastrostomy in ARPKD patients. Infection risk is an issue in patients on peritoneal dialysis (PD) (5, 6). If possible, gastrostomy insertion in PD patients should take place prior to or at the same time of PD catheter placement. If gastrostomy insertion becomes necessary after onset of PD, an open surgical procedure with protective sutures is recommended as opposed to the endoscopic technique (7). An increased risk of variceal bleeding in patients with portal hypertension and an increased risk of spleen injury in case of splenomegaly have been suggested for endoscopic gastrostomy insertion in patients with cystic fibrosis associated liver disease and portal hypertension (8) and patients with liver cirrhosis (9). Furthermore, the development of stomal varices after gastrostomy has been discussed (8, 10). ARPKD with accompanying portal hypertension and possible future liver transplantation has been classified as a relative contraindication for gastrostomy insertion in children by the French society of gastrointestinal endoscopy (11) and within the position paper of the European Society for Paediatric Gastroenterology, Hepatology, and Nutrition (ESPGHAN) on management of percutaneous endoscopic gastrostomy in children and adolescents (12).

To the best of our knowledge there are no previous studies addressing clinical approaches towards gastrostomy insertion and complications of gastrostomy in pediatric ARPKD patients. In order to gain insight into current practice, we conducted an anonymous web-based survey among pediatric nephrologists, pediatric hepatologists and pediatric gastroenterologists on their opinion and experiences concerning benefits, risks, and methods of gastrostomy insertion in ARPKD patients.

## Methods

### Study design, survey development, survey content, and administration

Questionnaires on the current clinical practice in gastrostomy insertion in patients with ARPKD (Supplementary data 1) were designed and validated by an expert group of pediatric nephrologists, hepatologists, and gastroenterologists. The survey was conducted in two steps: the first part was designed to assess general data on the background of participants as well as opinions regarding gastrostomy in patients suffering from CKD in general and ARPKD patients in particular. An additional aim was to identify specific conditions regarded as contraindications. The second part of the survey was designed to address gastrostomy insertion in ARPKD more specifically with respect to age at insertion, technique of insertion, periinterventional antibiotic and antifungal prophylaxis, signs of hepatic ARPKD involvement and conduction of dialysis at time of insertion. We specifically asked for observed complications after gastrostomy insertion in patients and for management of gastrostomy in case of subsequent transplantation. The survey ended with a personal evaluation of the risk-benefit analysis and the management of gastrostomy in non-ARPKD patients prior to transplantation.

The survey was an anonymous, web-based, cross-sectional study on a voluntary basis. Invitation to participate in the survey was sent to members of the European Society of Pediatric Nephrology (ESPN) study group, the International Pediatric Peritoneal Dialysis Network (IPDN) study group, the ARegPKD Consortium, the European Society for Paediatric Gastroenterology, Hepatology and Nutrition (ESPGHAN) Hepatology Interest Group, and the Pediatric Gastroenterology Internet Bulletin Board (PEDGI). The participants were asked to avoid duplicate entries in case of repeated invitation for the first part of the survey and to coordinate patient-specific entries within the own center in order to avoid multiple replies from a single center for the second part of the survey. The study was approved by the Ethics Committee of the Medical Faculty of the University of Cologne, Germany.

We received responses from 196 participants out of 39 countries with the largest groups of participants deriving from Germany (26), Poland (19), the United States of America (18), Belgium (17), the United Kingdom (14), France and Spain (each 11). Five or less participants each came from Argentinia, Australia, Austria, Brazil, Chile, China, Croatia, Denmark, Estonia, Georgia, Greece, Hungary, India, Iran, Kuwait, Lithuania, Macedonia, Netherlands, New Zealand, Oman, Pakistan, Peru, Portugal, Russia, Saudi Arabia, South Africa, South Korea, Sweden, Switzerland, and the United Arab Emirates. The profession of the participants was indicated as pediatric nephrologists (*n* = 141, 74.2%), pediatric hepatologists and/or gastroenterologists (*n* = 39, 20.5%), pediatrician (*n* = 3, 1.6%), pedatric nephrology/dialysis nurse (educator) (*n* = 2, 1.0%), dietitian (*n* = 2, 1.0%), and pediatric intensivist, adult nephrologist and trainee pediatric nephrology (each 1, each 0.5%). There were no replies by pediatric surgeons. 161/166 (97.0%) participants indicated that they took care of ARPKD patients in follow-up with a median number of 6 ARPKD patients (minimum one patient, maximum 60 patients). 152/164 (92.7%) participants performed pediatric PD and 145/164 (88.4%) performed hemodialysis (HD) at their institution. 118/162 (72.8%) participants performed kidney transplantation at their center, 65/159 (40.9%) performed liver transplantation, and 53/158 (33.5%) performed combined liver and kidney transplantation. 99/158 (60.7%) participants indicated that they had experience with management of variceal/portal hypertensive bleeding at their institution.

Patient-specific data was entered by 21 participants for a total of 38 patients. Most patients were from Germany, the United States of America, Denmark, and Iran. All patients showed specific characteristics in terms of country of origin, age, gender, and treatment modalities making duplicate entries of a single patient unlikely. Information regarding 32/38 (84.2%) patients was entered by pediatric nephrologists, for 3 (7.9%) patients information was given by pediatric hepatologists, information regarding one patient (2.6%) was entered by a pediatric gastroenterologist, pediatrician in training, and a nurse, respectively.

### Statistical analysis

Age at insertion of gastrostomy is given as median with interquartile range. All other variables were categorical and evaluated using descriptive statistics. Differences between nominal variables were calculated using Chi-Square test, significance was considered for *p* < 0.05. Data analysis was performed using IBM SPSS Statistics 22 for Windows.

## Results

### General attitude toward gastrostomy insertion in CKD and ARPKD

**Figure 1:**
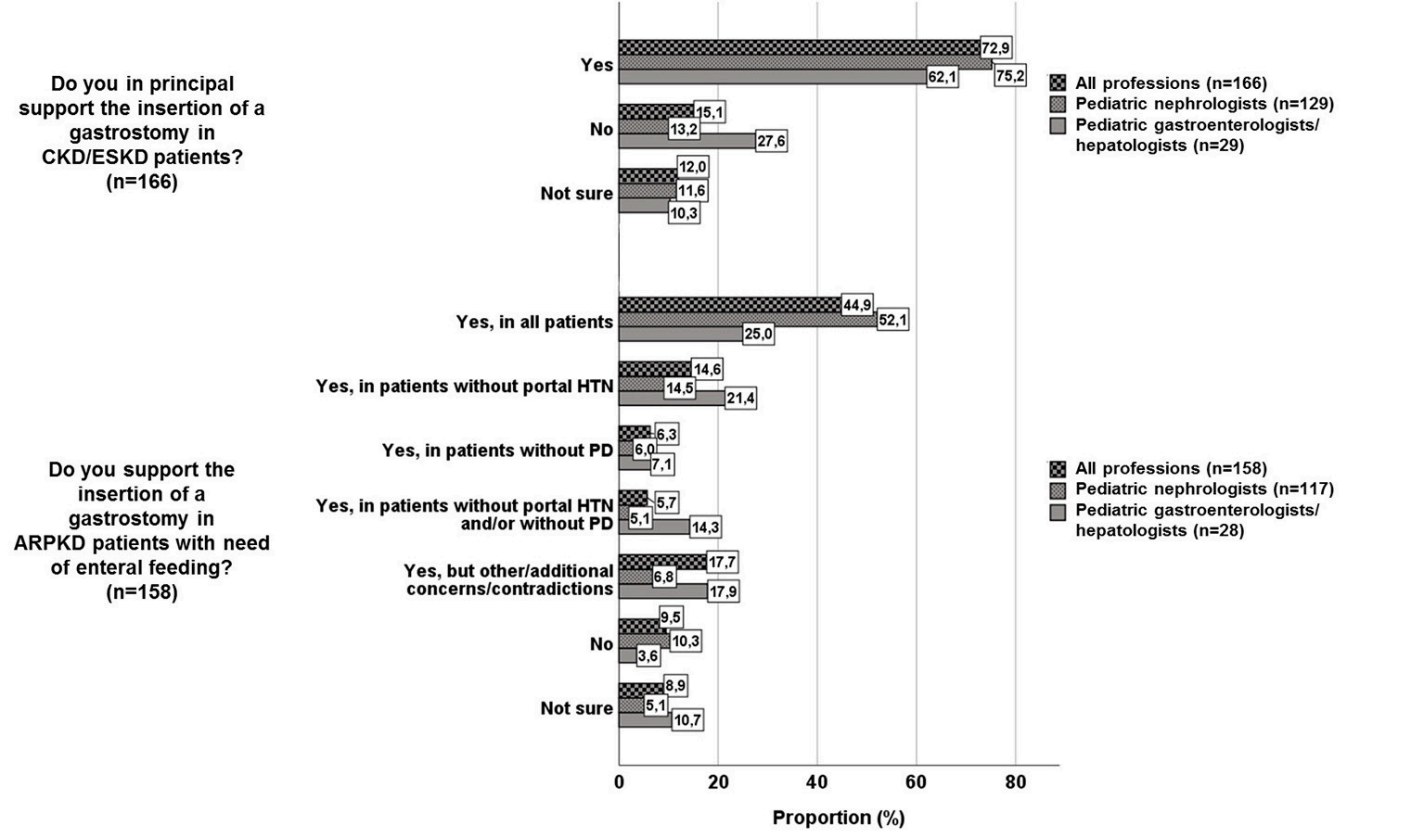
Support of gastrostomy insertion in CKD/ESKD patients and in ARPKD patients (HTN, hypertension; PD, peritoneal dialysis).

In principle, 121/166 (72.9%) participants supported gastrostomy insertion in patients with CKD and ESKD (Figure 1). Regarding gastrostomy insertion in ARPKD patients, participants showed more detailed answers: while 71/158 (44.9%) supported insertion in all ARPKD patients, 15/158 (9.5%) did not support gastrostomy insertion in ARPKD patients. 23/158 (14.6%) participants supported gastrostomy insertion only in ARPKD patients without signs of portal hypertension, 10/158 (6.3%) only in patients without PD and 9/158 (5.7%) only in patients without signs of portal hypertension and/or without PD (Figure 1). There were significantly more nephrologists [61/117 (52.1%)] supporting gastrostomy in all ARPKD patients compared to gastroenterologists/hepatologists [7/28 (25.0%), *p* = 0.01]. There was a trend to a more restrictive support of gastrostomy insertion in patients with signs of portal hypertension and/or PD in pediatric gastroenterologists/hepatologists compared to pediatric nephrologists (n.s., Figure 1). A substantial number of participants (28/158, 17.7%) raised other or additional concerns, that encompassed general factors (“precise case-by-case-decision,” “only in failure of all other techniques of feeding,” “fear of increased risk of severe local infections after transplantation,” “reluctance of surgeons or gastroenterologists to perform procedure”), PD-associated factors (“more strict indication in PD patients,” “only surgical procedure in case of PD”) and factors associated with portal hypertension (“more strict indication in case of portal hypertension,” “future liver transplantation as contraindication”) (Figure 1).

### Indications for gastrostomy insertion and hepatic involvement prior to insertion in ARPKD patients

**Figure 2:**
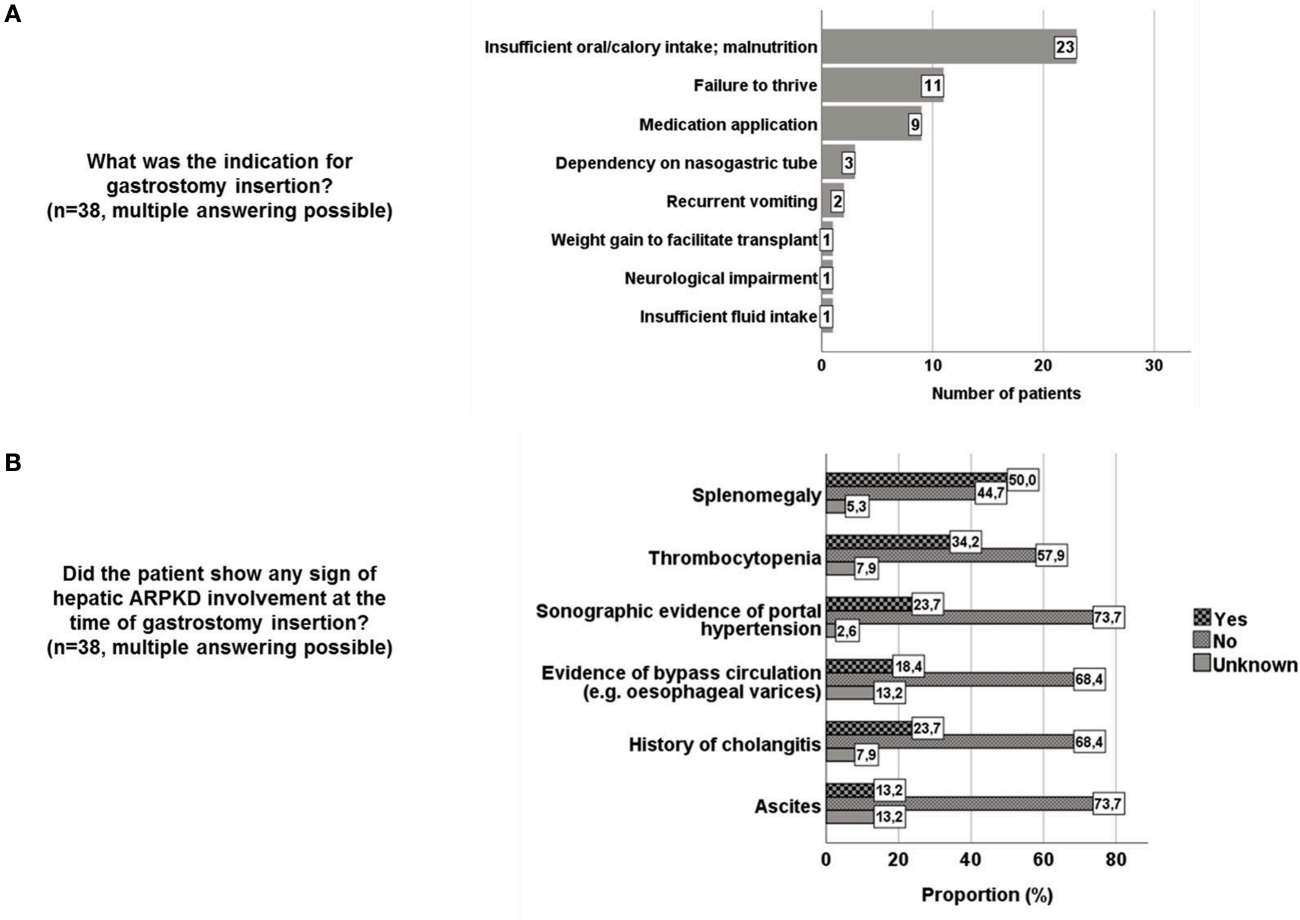
**(A)** Indications for gastrostomy insertion in 38 ARPKD patients. **(B)** Hepatic phenotype at time of gastrostomy insertion in 38 ARPKD patients.

Indications for gastrostomy insertion are shown in Figure 2A. Median age at insertion was 1.41 years (interquartile range 0.50–2.00 years). The youngest patient was 0.08 years, the oldest patient was 11.00 years old at gastrostomy insertion.

Prior to gastrostomy insertion more than half of the centers screened for varices in ARPKD patients (12/22, 54.5%). Most centers used esophagogastroduodenoscopy and/or ultrasound. Evaluation of the hepatic phenotype in specific patients indicated splenomegaly in 19/38 (50%) patients at time of gastrostomy insertion and evidence of collateral circulation (e.g., oesophageal varices) in 7/38 (18.4%) of patients (Figure 2B).

### Method of gastrostomy insertion in ARPKD patients

**Figure 3:**
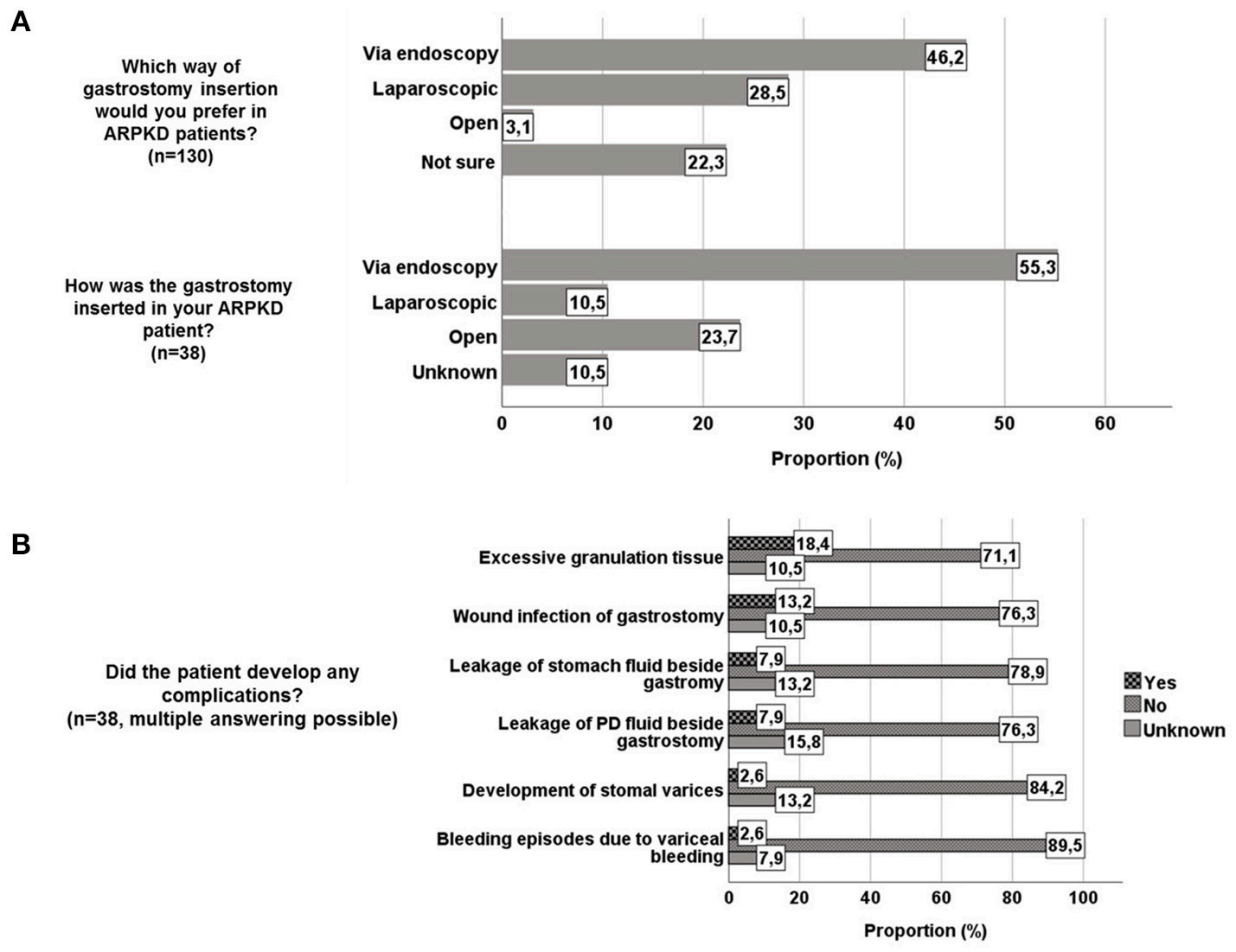
**(A)** Opinion regarding way of gastrostomy insertion in ARPKD patients and way of gastrostomy insertion in 38 ARPKD patients. **(B)** Reported complications of gastrostomy in 38 ARPKD patients.

In the identified 38 patients, gastrostomy was inserted via endoscopy in 21/38 (55.3%), laparoscopically in 4/38 (10.5%), and in an open surgical approach in 9/38 (23.7%) patients. This differs from the opinion indicated in the first part of the survey, in which participants reported to favor insertion via endoscopy (60/130, 46.2%) or laparoscopic (37/130, 28.5%), while open insertion was preferred by only 4/130 (3.1%) participants with 29/130 (22.3%) participants being unsure about their preference in ARPKD patients (Figure 3A). The preference of different methods of insertion did not differ between pediatric gastroenterologists/hepatologists and nephrologists. In general CKD/ESKD patients, 72/117 (61.5%) participants favored endoscopic insertion, 25/117 (21.4%) laparoscopic, and 2/117 (1.7%) open insertion, while 18/117 (15.4%) were unsure about their preferred method.

### Complications of gastrostomy in ARPKD patients

Reported complications of gastrostomy insertion encompassed excessive granulation tissue (7/38, 18.4%) and wound infection (5/38, 13.2%). In 3/38 (7.9%) patients each leakage of PD fluid or stomach fluid through gastrostomy was reported. One patient each (2.6% each) developed stomal varices, bleeding episodes due to variceal bleeding and excessive bleeding from gastrostomy exit site 9 months after insertion (Figure 3B). Other complications encompassed buried bumper, hernia, requirement of surgical closure after gastrostomy removal and a suspected association with a recurrent *Clostridium difficile* infection. One patient was reported to have suffered from preinterventional hypotension during anesthetic induction with subsequent cerebral infarction. 5/38 (13.2%) patients required surgical revision (change to button, herniotomy, surgical closure, due to infection), including one patient who was reported to have developed fulminant sepsis with subsequent death. Detailed information regarding causality and temporal connection of gastrostomy with fulminant sepsis was not available due to the anonymous setting of the survey. 7/52 (13.5%) colleagues who responded to the general questionnaire indicated the requirement for gastrostomy removal in ARPKD patients for various reasons (gastrocutaneous fistula, infection, discomfort of patient resp. parents'refusal of continuation, pretransplant removal due to sufficient weight gain).

### Antiinfectious prophylaxis

Prophylactic antibiotics were applied to all patients undergoing gastrostomy insertion in 8/22 (36.4%) centers, while 3/22 (13.6%) centers indicated prophylactic antibiotics only in ARPKD patients. No prophylactic antibiotics were given in 7/22 (31.8%) centers. Cephalosporines were most frequently used [8/10 (80.0%) centers]. 2/22 (9.1%) centers used prophylactic antifungals (fluconazole) in both ARPKD and non-ARPKD patients.

### Dialysis and transplantation

10/38 (26.3%) patients with gastrostomy already performed PD at time of gastrostomy insertion. 4/38 (10.5%) patients performed HD at time of gastrostomy insertion. 13/38 patients (34.2%) started PD after gastrostomy insertion, 2/38 (5.3%) patients started HD after gastrostomy insertion.

18/38 (47.4%) patients underwent transplantation with inserted gastrostomy (10 kidney transplantations, 8 combined liver and kidney transplantations). Gastrostomy was removed at timepoint of transplantation in 3 cases (one patient with kidney transplantation from one center in Iran, two patients with combined liver and kidney transplantation from one German center) and was kept in all other patients. In non-ARPKD patients gastrostomy was removed in 6/64 (9.4%) centers prior to transplantation. One colleague reported that gastrostomy is removed in non-ARPKD patients in case of liver, but not in case of kidney transplantation.

### General evaluation of gastrostomy in ARPKD patients

63/158 (39.9%) participants indicated at least one ARPKD patient who had undergone gastrostomy insertion at their center with 7/65 (10.8%) colleagues recalling significant complications. Yet, in summary, 58/64 (90.6%) participants retrospectively summarized that gastrostomy insertion was the right decision for their patients, including 12/15 (80.0%) pediatric gastroenterologists/hepatologists, and 42/45 (93.3%) pediatric nephrologists (*p* = 0.14).

In summary 34/38 (89.5%) patients were considered to benefit from gastrostomy with respect to development and growth. Gastrostomy insertion was evaluated as right decision for the patient in 35/38 (92.1%) patients. In two cases gastrostomy insertion was interpreted as not beneficial for the patient, with one patient developing preinterventional hypotension with neurological sequelae and one patient dying from fulminant sepsis with unclear causality or temporal connection to gastrostomy insertion.

## Discussion

Based on an online survey we report on the first data on current practice of gastrostomy insertion in patients with ARPKD. To the best of our knowledge, this pediatric ARPKD cohort is the first to be reported with details on method of insertion and associated complications.

When evaluating the opinion in a first survey with 196 participants, ARPKD was considered to be a special condition in comparison to other CKD/ESKD causes. Both portal hypertension and PD were mentioned as (relative) contraindications for gastrostomy in ARPKD patients. This reflects the presumption of these two conditions as precautions for gastrostomy insertion in ARPKD patients (5, 7, 11, 12). Interestingly, pediatric gastroenterologists/hepatologists seemed to be more cautious in inserting gastrostomy in comparison to pediatric nephrologists. These differences in opinions may point out the need of a multidisciplinary discussion of both indications and contraindications between pediatric nephrologists and gastroenterologists/hepatologists to offer a uniform concept to affected patients and their families.

The common indications of insertion in 38 ARPKD patients encompassed malnutrition, failure to thrive and safe medication administration. Weight gain can be a major challenge in CKD and ARPKD (6, 13), but is substantial for achievement of a sufficient body weight for transplantation (about 10 kg in many centers). After kidney transplantation, a safe way of medication and fluid application via gastrostomy can facilitate management. Importantly, the indication for gastrostomy tube insertion in a specific ARPKD patient needs to be assessed on a case-by-case basis implementing the aspects of malnutrition as well as the renal, hepatic and neurological phenotype of an individual patient.

Our series does not report greatly increased proportions of complications in ARPKD patients compared to other pediatric patients. The most frequent reported complications were excessive granulation tissue and wound infection not exceeding complication rates in larger series of children with percutaneous endoscopic or laparoscopic gastrostomy (12, 14–16). Two cases reported in our study deserve a closer look: in one patient who had undergone bilateral nephrectomy and who suffered from severe blood pressure variations, arterial hypotension developed during anesthetic induction for gastrostomy insertion. This lead to sequelae of neurological impairment and the attending physician's assessment that the gastrostomy insertion was the wrong decision for this specific patient. However, the reported complication may appear to be related rather to the general risks of anesthesia after bilateral nephrectomy than to the specific intervention of gastrostomy insertion. In another case, a colleague reported fulminant sepsis with death in an APRKD patient. From our data, we cannot specify whether this event was directly linked to gastrostomy insertion, as detailed information on this case was not available in our anonymous study.

Regarding the hepatic phenotype of patients requiring gastrostomy, conditions of portal hypertension and/or possible future liver transplantation are considered as relative contraindications for percutaneous endoscopic gastrostomy (PEG) insertion by the ESPGHAN (12). The position paper mentions the risk of *de novo* portosystemic shunts and peristomal varices which could cause severe bleeding and pose major challenges for future liver transplantation. Very limited evidence of similar scenarios dealing with portal hypertension in patients with PEG are cited from the literature (12) in form of only two studies with 2 respectively 5 patients suffering from portal hypertension receiving PEG (17, 18). The authors underline that careful preparation and adequate expertise are mandatory requirements for PEG insertions in these specific patients. In our survey, surprisingly, half of the 38 ARPKD patients showed some form of hepatic involvement/phenotype at the time of gastrostomy insertion. Apparently, the benefits of gastrostomy insertion were considered to outweigh potential complications in these patients. Bleeding-related complications were reported in 3 of 38 patients (7.9%): one patient each was reported to suffer from stomal varices respectively bleeding episodes due to variceal bleeding summing up to two patients (5.3%) with portal hypertension related bleeding complications. The third patient (1 of 38, 2.6%) was reported to suffer from excessive bleeding from gastrostomy exit site 9 months after insertion. It remained unclear whether this bleeding episode was related to portal hypertension or rather to gastrostomy tube induced mucosal irritation. In the literature, data are very limited regarding bleeding complications in pediatric patients with portal hypertension and gastrostomy. First data exist for children with cystic fibrosis associated liver disease: two case series with 7 and 37 patients report no bleeding episodes attributable to varices or development of stomal varices, minor complications in common frequency and no procedure-related mortality (8, 10). Compared to this, the rate of complications related to portal hypertension in our series may seem to be relevant and needs to be taken into consideration in the decision process prior to gastrostomy insertion. Assuming that the patient with excessive bleeding from gastrostomy exit site 9 months after insertion suffered from tube induced mucosal irritation, this rate seems to be within the same range of a single center experience from South Korea (19). In this study on 236 pediatric non-ARPKD patients undergoing gastrostomy insertion due to poor nutrition, swallowing difficulty, and upper gastrointestinal obstruction gastrointestinal (GI) bleeding due to gastrostomy irritation was reported in up to 5.4% of their cohort (19).

The ESPGHAN position paper on management of PEG insertion in children and adolescents does not indicate any evidence regarding the management of gastrostomy tubes in patients with potential upcoming liver transplantations (12). Almost half of the patients (18/38) with inserted gastrostomy in our dataset underwent transplantation with a gastrostomy in situ. Ten patients received isolated kidney transplantation and 8 patients received combined liver and kidney transplantation. In three patients (two with combined liver and kidney transplantation, one with isolated kidney transplantation), gastrostomy was removed at the timepoint of transplantation. No adverse events or severe complications were reported in the courses after transplantation. As there is no recommendation regarding timepoint of gastrostomy removal in children after transplantation, risks and benefits need to be outweighed in every single patient (20, 21). Our data without severe complications in the post-transplant courses in 15 ARPKD patients with gastrostomy in situ (9 patients with isolated kidney transplantation, 6 patients with combined liver and kidney transplantation) may set a basis for discussing the timepoint of gastrostomy removal.

Limitations of our survey include the anonymous questionnaire which does not allow queries for both participants and survey organizers in case of uncertainties. The set-up of the survey may result in a selection bias of centers. We can neither exclude a bias in participation due to personal experiences nor a bias in reporting in both positive and negative aspects. Due to pre-determined answer possibilities, specifications were not possible in all questions. Answers of questions might be biased to a nephrologic point of view, since three quarter of all participants indicated to be pediatric nephrologists and only 20% indicated to be pediatric gastroenterologists/hepatologists. We did not receive replies from pediatric surgeons potentially precluding addition of information from a surgical point of view. On the other hand, regular patient care and follow-up is provided by pediatric nephrologists and gastroenterologists/hepatologists in most pediatric ARPKD patients. Furthermore, pediatric gastroenterologists/hepatologists take care of gastrostomy insertion and follow-up in the first place in many centers consulting pediatric surgeons only in case of uncertainties or contraindications for non-surgical insertion. As further limitation, patient-specific data did not encompass a longitudinal follow-up of data on growth and development.

The choice of insertion method in complex patients—such as children with ARPKD—is subject to local habits and experiences at specific centers. Since the cohort of pediatric ARPKD patients displays major phenotypic variability with respect to growth and development as well as renal and hepatic phenotype, the results of our survey can neither be extrapolated to daily clinical practice without critical case-by-case discussion in a multidisciplinary team nor replace future clinical practice recommendations or guidelines.

Despite these limitations, this study sets a first basis of reporting encouraging international experiences with gastrostomy insertion in pediatric ARPKD patients. In summary, gastrostomy insertion was evaluated as a correct decision for the patient and outweighing developing complications in most cases. Due to the concomitant hepatorenal affection, children with ARPKD rely on a multidisciplinary collaboration of both pediatric nephrologists and gastroenterologists/hepatologists (22). In order to set a basis for development of management recommendations in a multidisciplinary approach, international initiatives like the recently established ARPKD registry Study ARegPKD will further help to define indications and contraindications of gastrostomy insertion in ARPKD patients (23, 24).

## Author contributions

KB, JB, RS, PW, LW, JD, FS, DM, and ML drafted the manuscript. KB, JB, RS, PW, FS, DM, and ML designed the questionnaire. All authors reviewed and approved the final manuscript.

## Data availability

The raw data supporting the conclusions of this manuscript will be made available by the authors, without undue reservation, to any qualified researcher.

### Conflict of interest statement

The authors declare that the research was conducted in the absence of any commercial or financial relationships that could be construed as a potential conflict of interest.

## Abbreviations

ARPKD: autosomal recessive polycystic kidney disease
CHF: congenital hepatic fibrosis
CKD: chronic kidney disease
ESKD: end-stage kidney disease
GI: gastrointestinal
HD: hemodialysis
HTN: hypertension
n.s.: not significant
PD: peritoneal dialysis
PEG: percutaneous endoscopic gastrostomy.
