# Supplementary material for: Gastrostomy Tube Insertion in Pediatric Patients With Autosomal Recessive Polycystic Kidney Disease (ARPKD): Current Practice
Source: Front Pediatr. 2018 Jun 4;6:164. doi: 10.3389/fped.2018.00164 (PMC5994991; doi:10.3389/fped.2018.00164)
Supplement: Supplementary file 1 [file Data_Sheet_1.pdf]

Supplementary data 1

Questionnaires on the current clinical practice in gastrostomy insertion in patients with Autosomal recessive polycystic kidney disease (ARPKD)

Part 1

1. In which country is your department located?
2. Please choose your profession: Pediatric nephrologist - Pediatric hepatologist - Surgeon - Other (please specify)
3. How many pediatric ARPKD patients are currently in follow-up in your department?
4. At your institution do you perform…:
    - Pediatric peritoneal dialysis? Yes - No - Not sure
    - Pediatric hemodialysis? Yes - No - Not sure
    - Pediatric renal transplantation? Yes - No - Not sure
    - Pediatric liver transplantation? Yes - No - Not sure
    - Pediatric combined liver and kidney transplantation? Yes - No - Not sure
    - Frequent management of variceal and portal hypertensive bleeding? Yes - No - Not sure
5. Do you in principal support the insertion of a gastrostomy in CKD/ESRD patients? Yes - No - Not sure
    - If yes: Which way of gastrostomy insertion would you prefer in CKD/ESRD patients? Open - Laparoscopic - Via endoscopy - Not sure
6. Do you support the insertion of a gastrostomy in ARPKD patients with need of enteral feeding? Yes, in all the patients - Yes, in patients without portal hypertension - Yes, in patients without peritoneal dialysis - No, I do not support the insertion of a gastrostomy - Not sure - Yes, but other concern/contradictions
    - If yes: Which way of gastrostomy insertion would you prefer in ARPKD patients? Open - Laparoscopic - Via endoscopy - Not sure
7. Do you remember ARPKD patients in your centre who had a gastrostomy inserted? Yes - No - Not sure
    - If yes: Do you remember significant complications regarding gastrostomy insertion in ARPKD patients? Yes - No - Not sure
        - If yes: Please specify the complications you remember
    - If yes: Do you remember having to remove a gastrostomy in ARPKD patients? Yes - No - Unknown
        - If yes: For which reasons did you have to remove the gastrostomy?
    - If yes: Would you retrospectively summarize gastrostomy insertion was the right decision for your patient(s)? Yes - No - Unknown
        - If no: Please indicate reasons why you think gastrostomy insertion was not the right decision for your patient(s)
8. In a second survey, we will obtain patient-specific data in more detail. We would appreciate your contribution. Please indicate whether you would be willing to share your experiences in such a project. Yes, I am interested. - No, I am not interested.

Part 2

1. In which country is your department located?
2. Please choose your profession: Pediatric nephrologist - Pediatric hepatologist - Surgeon - Other (please specify)
3. Has any of your ARPKD patients ever had a gastrostomy inserted? Yes - No - Unknown
    - If yes: How many patients had a gastrostomy inserted? 1 - 2 - 3 - 4 - 5
4. With respect to specific patient:
    - At what age was the gastrostomy inserted?
    - What was the indication for gastrostomy insertion?
    - How was the gastrostomy inserted? Open - Laparoscopic - Via endoscopy - Unknown
    - Did the patient show any sign of hepatic ARPKD involvement at the time of insertion?
        - Splenomegaly: Yes - No - Unknown
        - Thrombocytopenia: Yes - No - Unknown
        - Sonographic evidence for portal hypertension: Yes - No - Unknown
        - Evidence of bypass circulation (e.g. oesophageal varices): Yes - No - Unknown
        - History of cholangitis: Yes - No - Unknown
        - Ascites: Yes - No - Unknown
    - Was the patient on PD/HD at the time of insertion? Yes, the patient was on PD at time of insertion - Yes, the patient was on HD at time of insertion - No - Unknown
    - Was your patient with inserted gastrostomy taken on PD/HD? Yes, the patient was taken on PD - Yes, the patient was taken on HD - No - Unknown
    - Did the patient develop any complications?
        - Leakage of PD fluid beside gastrostomy: Yes - No - Unknown/Does not apply
        - Leakage of stomach fluid beside gastrostomy: Yes - No - Unknown/Does not apply
        - Wound infection of gastrostomy: Yes - No - Unknown/Does not apply
        - Excessive granulation tissue: Yes - No - Unknown/Does not apply
        - Development of stomal varices: Yes - No - Unknown/Does not apply
        - Bleeding episodes due to variceal bleeding: Yes - No - Unknown/Does not apply
        - Others (please specify)
    - Was there any need for revision surgery of gastrostomy? Yes - No - Unknown
        - If you answered “yes” to the previous question, please specify the surgery needed
    - Did the patient receive isolated kidney Tx or isolated liver Tx or combined liver kidney Tx with inserted gastrostomy? Yes, isolated kidney Tx - Yes, isolated liver Tx - Yes, combined liver kidney Tx - No - Unknown
        - If your patient received a Tx, was the gastrostomy removed at the time of Tx? Yes - No - Unknown
    - Did the patient - in your opinion - benefit from gastrostomy with regard to weight development and growth? Yes - No - Unknown
    - Would you retrospectively summarize gastrostomy insertion was the right decision for your patient? Yes - No - Unknown
        - If no: please indicate reasons
5. In general, do you give prophylactic antibiotics at time of insertion of a gastrostomy in ARPKD and non-ARPKD patients? Yes, in ARPKD and non-ARPKD patients - Yes, only in ARPKD patients, Yes, only in non-ARPKD patients - No - Unknown
    - If yes: Which antibiotic do you give prophylactically?
6. In general, do you give prophylactic antifungals at time of insertion of a gastrostomy in ARPKD and non-ARPKD patients? Yes, in ARPKD and non-ARPKD patients - Yes, only in ARPKD patients, Yes, only in non-ARPKD patients - No - Unknown
    - If yes: Which antifungal do you give prophylactically?
7. Do you screen for varices before gastrostomy insertion in ARPKD patients? Yes - No - Unknown

- If yes: How do you screen for varices?

8. Do you remove a gastrostomy in other patients (not ARPKD) prior or during transplantation (kidney or liver or combined liver kidney)? Yes - No - Unknown
    - If yes: For which reason do you remove a gastrostomy in other patients prior or during transplantation (kidney or liver or combined liver kidney)?
9. Did you ever have to remove a gastrostomy in an ARPKD patient? Yes - No - Unknown
    - If yes: For which reason did you have to remove a gastrostomy in ARPKD patients?
